# Factors determining curtailment behaviour of youths: moderating role of government policies

**DOI:** 10.3389/fpsyg.2024.1332422

**Published:** 2024-08-16

**Authors:** Asad Ahmad, Swati Garg, Mohd Danish Kirmani, Dag Øivind Madsen

**Affiliations:** ^1^Department of Management, Jamia Hamdard, New Delhi, India; ^2^School of Management, IILM University, Greater Noida, India; ^3^Department of Business, Marketing and Law, USN School of Business, University of South-Eastern Norway, Hønefoss, Norway

**Keywords:** curtailment behaviour, eco-concern, financial concern, government policy, religiosity, social norms

## Abstract

**Introduction:**

Environmental degradation poses a significant threat, making the comprehension of sustainable behaviour imperative for both environmental and business reasons. Embracing sustainable practices and reducing the unnecessary consumption of resources is essential in our current times. One can be sustainable by focusing on either buying sustainable or green products or by using fewer resources, i.e., by adopting curtailment behaviour. The purpose of this study is to determine various factors and their impact on the curtailment behaviour of youths.

**Methods:**

We used an exploratory and descriptive research design. Responses were generated from 513 young respondents using a structured questionnaire developed based on the extant literature.

**Results:**

The structural model findings showed that social norms, eco-concern, and religiosity significantly and positively affect curtailment behaviour. However, we observed no significant influence of financial concern or government policy on curtailment behaviour. We also tested the moderation impact of government policy on the relationship between economic concern and curtailment behaviour. The findings suggest that the relationship between eco-concern and curtailment behaviour is stronger for consumers exhibiting higher commitment to government policy.

**Discussion:**

It is imperative that politicians exercise critical thought and devise strategies to encourage more sustainable consumer behaviour. With the strains that our world is under now and in the future, we need to unite around a common goal: ensuring that our planet is sustainable for coming generations. The study findings are useful for academicians, marketers, and policymakers.

## 1 Introduction

The true wealth of a nation lies in its available resources. But what happens when those resources are scarce relative to the population? The world’s population is increasing at such a great pace that we are unable to increase our resources in the same proportion. Moreover, carbon footprints are increasing day by day, so to preserve the environment, the only option we have is to act sustainably. The ability to live and flourish without affecting natural resources for the future is referred to as sustainability. Sustainable behaviour can be implemented in two ways: either by using efficient or eco-friendly resources or by curtailing the usage of existing resources. Our consumption habits have prompted both societies and businesses to face a myriad of challenges, signalling a shift toward a new business paradigm. This shift is underscored by environmental degradation, pollution, the increase in extreme weather events, the widening gap of social inequality, and the rise in poverty, along with an escalating demand for renewable energy sources. This confluence of issues underscores the urgent need for sustainable practices and innovations in how we produce, consume, and manage resources (White et al., 2019).

As responsible citizens, we have a duty to conserve resources by either using them to their maximum efficiency or by reducing their use. It can be done through sustainable behaviour and/or curtailment behaviour. When a consumer changes his or her daily habits or makes compromises to save the environment, this behaviour can be described as a practice of curtailment. With growing concerns regarding the environment, the economy, and resource depletion, it has remained a persistent topic on the political agenda (Karlin et al., 2012). For instance, there have been policies for smart charging plug-in electric vehicles (PEVs), which may help energy curtailment by up to 40% compared to unmanaged PEVs (Szinai et al., 2020). Curtailment is part of sustainability, and although the distinction between efficiency (sustainability) and curtailment behaviour is not novel (Umit et al., 2019), several researchers have used sustainable behaviour synonymously with curtailment behaviour. Better energy management behaviour is *“putting a cover on a pot while boiling water*” and curtailment behaviour is “*wearing a sweater instead of turning up the heater in winter*” (Van Den Broek et al., 2019).

Pro-environmental self-identity (PESI) has a direct impact on pro-environmental behaviour. PESI helps in understanding the sustainable consumption behaviour of pro-environmental customers (at the individual or group level) who engage in both buying and curtailment behaviours. Thus, pro-environmental behaviour means engaging in curtailment behaviour. Curtailment behaviour appears to be more reliable and practical in handling environmental issues, but it is comparatively difficult to implement since it requires sacrifices on the part of individuals (Garg et al., 2023). Such behaviour often has minimal or no monetary costs but may have significant non-monetary costs, for example, in terms of time, effort, knowledge, and inconvenience (De Nardo et al., 2017). Consumers can make a great difference in resolving environmental conditions through their consumption choices and behaviour. Consumers may engage in pro-environmental behaviour in a variety of ways, including recycling products (Onel and Mukherjee, 2017), conserving energy, and bill consciousness (Karlin et al., 2012).

Particularly, young consumers have demonstrated a pronounced concern for health and environmental factors when making purchasing decisions (Lago et al., 2020). Prieto-Sandoval et al. (2022) similarly identified a strong inclination towards sustainable behaviour among this demographic. Numerous other studies (e.g., Haug and Hassinggaard, 2023; Rossi and Rivetti, 2023; Su et al., 2023) corroborate the sustainable tendencies of young consumers. Consequently, it is anticipated that young consumers will actively participate in curtailment behaviour to conserve environmental resources. Testa et al. (2016) previously confirmed the willingness of young consumers to engage in such behaviour. A comprehensive examination of curtailment behaviour among young consumers is warranted for two primary reasons. Firstly, The proportion of youth (around 30%) in the Indian population is the highest in the world (Youth in India, 2022). Secondly, young consumers play a pivotal role in family decision-making (Chavda et al., 2005; Ashraf and Khan, 2016; Chaudhary, 2018; Kirmani et al., 2020), positioning them as potential advocates for curtailment behaviour within their households. Further, the adoption of curtailment behaviour by young consumers could establish a positive trend for subsequent generations.

Based on previous research, the two types of behaviour changes are efficiency behaviour (Never et al., 2022) and curtailment behaviour (Testa et al., 2016). While there are various studies on efficiency behaviour, very few studies have been done on curtailment behaviour. Efficiency behaviour refers to occasional and expensive behaviour that requires spending money on efficient technologies such as LED, whereas curtailment behaviour refers to using the existing technologies less and requires us to forego comfort or luxuries, e.g., switching off the lights when not in use (Umit et al., 2019). Curtailment behaviour has also been known as “habitual behaviour, long-term habits, or repetitive behaviour.” Curtailment behaviour is pro-social in nature (Gandhe and Pandey, 2017), where rather than purchasing a specific product, the reduction or modification of the goods or service is typically achieved through behavioural change (De Nardo et al., 2017). Everyday actions that reduce carbon footprints can have a significant impact on the environment if they are combined. Households are not required to spend money on curtailment efforts, but they must invest time in them (Matsumoto and Sugeta, 2022).

Given the pressing circumstances, it is crucial that we adopt sustainable behaviours and reduce the unnecessary consumption of resources. Businesses may aim for consumers to acknowledge, appreciate, and reward their sustainable values and actions in ways that drive sustainable consumption and enhance the firm’s sustainability and strategic economic benefits as they operate and deliver products and services more sustainably (White et al., 2019). Concern and proper understanding of environmental issues are referred to as environmental awareness. Achieving sustainability requires focusing on one or more of the following strategies. First, buying sustainable or green products, which indirectly helps reduce harmful impacts on the environment, or second, using fewer resources, i.e., by adopting curtailed behaviour. Both types help with sustainability in their own way. Irrespective of the relevance of curtailment behaviour, there is a lack of studies in this area. Therefore, in the current study, we focus on identifying the diverse factors influencing curtailment behaviour among young people, who represent the future of every nation.

## 2 Conceptual framework

In an age marked by environmental degradation, our economies must adopt more sustainable practices. Adopting curtailment behaviour can reduce consumption patterns and also lead to financial savings compared to regular usage (Sudarmaji et al., 2022). It may include minimising appliance usage, disconnecting appliances, and switching off the lights. To adopt or promote such behaviour, we need to have a proper understanding of the ground realities and the factors that influence it. To explore the determinants of curtailment behaviour, researchers in previous studies have consulted various bodies of literature. Curtailment is something that is related to demand-side management (Garg et al., 2023). One of the demand-side management programmes that lower energy usage and emissions is energy efficiency (Sudarmaji et al., 2022).

**Figure 1:**
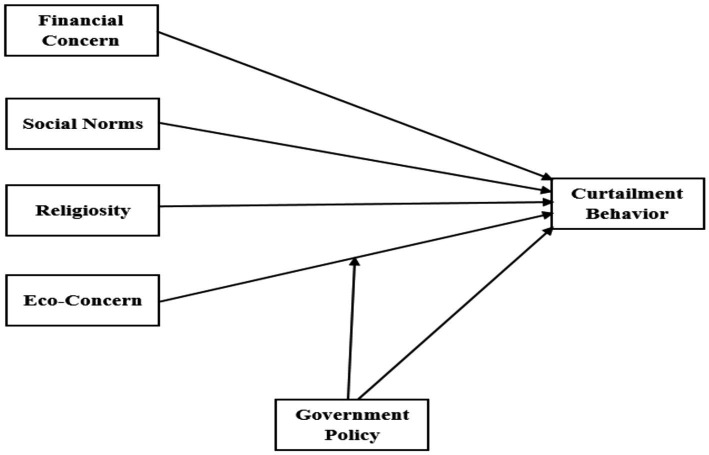
Proposed model.

Consumer research on curtailment behaviour is sparse. Nonetheless, a few scattered studies have endeavoured to identify the factors influencing consumers’ curtailment behaviour. However, most of those studies have focused on household energy conservation (Umit et al., 2019; Never et al., 2022). Every electrical curtailment behaviour is influenced by a unique set of variables, with age, gender, and perceived behavioural control being statistically significant predictors. Taking cues from the extant literature, a model (Figure 1) is proposed, comprising factors determining curtailment behaviour.

### 2.1 Financial concern and curtailment behaviour

People appear to be concerned about energy consumption for financial reasons (Testa et al., 2016). On the one hand, habitual practices (like walking or cycling for short distances instead of using a car) need no or little initial investment, making them especially appealing to low-income people (Umit et al., 2019). Saving by cutting back, on the other hand, is dependent on breaking behaviours associated with comfort and social standing (Adnan et al., 2023). While efficiency behaviours normally necessitate substantial investments, curtailment behaviours necessitate more effort and limit people’s flexibility to travel (Schuitema and Bergstad, 2019). Financial discounts are typically regarded as sufficient compensation for the inconvenience caused by the curtailment (Curtis et al., 2020). Consumer purchasing power, or the ability to acquire things, fosters environmental awareness and creates a potential market for sustainable goods (Garg et al., 2023). Studies demonstrate a favourable relationship between financial concerns and the practise of energy conservation (Chen et al., 2017). Many researchers show that financial benefits from lower operational costs, higher fuel efficiency, or other secondary expenditures influence consumers’ willingness to purchase environmentally friendly products (Huang et al., 2018). Consequently, the following hypothesis was proposed:

*H1*: Financial Concern positively impacts the Curtailment Behaviour of an individual.

### 2.2 Social norms and curtailment behaviour

Social norms, which can be classified into two categories: descriptive norms and injunctive norms, play a significant role in curtailment behaviour (Karlin et al., 2012). It is described as a belief about whether a particular behaviour would be approved by a person’s social environment (Onel and Mukherjee, 2017). Those who claim that curtailment is essential for long-term sustainability must confront status attitudes, as social stigmas may prevent them from being adopted (De Nardo et al., 2017). Social pressure, i.e., the fear of social punishment, is thought to validate the behavioural impact of social norms. One convincing line of reasoning that was prominently absent from previous studies was based on curtailment behaviour and social norms. One study’s findings have also raised concerns that perhaps social norms were not successfully utilised or were not a major driver of energy consumption or curtailment behaviour (Van Den Broek et al., 2019). But some studies suggest social norms have a strong effect on individuals’ pro-social and pro-environmental behaviour, which is a curtailment behaviour (Reese et al., 2014). Taking into account the significance of social norms, the following hypothesis was subsequently formulated:

*H2*: Social Norms positively impact the Curtailment Behaviour of an individual.

### 2.3 Religiosity and curtailment behaviour

Religiosity is an individual’s belief in God and dedication to his faith (Kirmani and Khan, 2016). Religion has a large influence on people’s views, beliefs, and behaviour; thus, it needs proper concern as an important cultural aspect. Over two-thirds of the world’s population considers religion to be important in their everyday life decisions, influencing their beliefs, attitudes, and actions (Abutaleb et al., 2020). Religious feelings have been positively related to a stronger sense of individual duty to safeguard the environment and adversely related to NEP (New Environmental Paradigm) support (Jurdi-Hage et al., 2019). In one study, it was observed that those who scored high on the religiosity scale were less spontaneous when deciding what to buy (Koku and Jusoh, 2014). Religious principles are closely related to a natural environmental perspective and environmental awareness. The impact of religiosity on consumer behaviour and purchasing decisions has revealed that a particular segment of consumers spend conservatively even when they have the means to spend lavishly because they follow the commandments of their lord (Alam et al., 2011). Consumers with higher levels of inherent religious orientation are more likely to buy eco-friendly goods, but they do not show curtailment behaviour. People can easily evaluate and assess problems in their lives using the structure that religion offers (Abutaleb et al., 2020). Several studies have connected religiosity to a variety of values and behaviours, including pro-environmental values, attitudes, and behaviours (Clements et al., 2014). Considering the aspect of religiosity, the subsequent hypothesis was proposed:

*H3*: Religiosity positively impacts the Curtailment Behaviour of an individual.

### 2.4 Eco-concern and curtailment behaviour

When people expect that environmental policies will lessen societal difficulties, they are more likely to support them (Suki, 2016). To increase the acceptability of environmental policies, one way is to compensate citizens for the negative repercussions of these policies, which can be done by enacting a package of policy measures rather than single policies (Schuitema and Bergstad, 2019). The number of products advertised as environmentally friendly has significantly increased because of growing environmental concerns, which seem to benefit all levels of society. The environmental benefits of conservation behaviour may be more effective at reminding people to engage in curtailment activities, whereas appeals focused on overcoming real and/or perceived barriers to activity (e.g., cost, self-efficacy) may be more effective at promoting efficiency behaviours (Karlin et al., 2012). Recent studies focus on understanding the impact of eco-concern on curtailment behaviour as a sustainable approach.

Thus, the following hypothesis was proposed:

*H4*: Eco-concern positively impacts the Curtailment Behaviour of an individual.

### 2.5 Government policy and curtailment behaviour

People who care about the environment tend to perceive that “environmental protection is a governmental duty” (Chen et al., 2018). Researchers have suggested that government policies play a significant role in determining the attitudes of consumers. Tariffs imposed by the government can have a considerable impact on domestic energy conservation (Prasanna et al., 2018). However, the direct impact of government policies on residents’ procurement of energy-efficient products and daily energy-saving behaviour, as well as the interaction of government policies and other factors influencing household energy-saving behaviour, remains to be adequately demonstrated (Hong et al., 2019). To encourage the use of energy-efficient products, the government provides subsidies and price recommendations.

Therefore, the following hypothesis was proposed:

*H5*: Government Policy positively impacts the Curtailment Behaviour of an individual.

### 2.6 Moderating role of government policy on eco-concern and curtailment behaviour

Several studies suggest the positive impact of government policies on green buying behaviour (Chen et al., 2018). Governmental policies are instrumental in predicting the environmental attitudes of consumers. Environment-friendly people strongly believe that protecting the environment is one of the most important responsibilities of any government. The existing literature suggests several researchers attempting to find out the direct impact of government policies on sustainable behaviour, but few studies have tried to find out the interaction between government policies and other factors determining sustainable behaviour (Chen et al., 2018). Drawing on findings from existing research, we propose that government policies can act as a guiding principle for consumers to be environmentally concerned. Thus, we proposed the following hypothesis:

*H6*: Government Policy positively moderates the impact of Eco Concern on the Curtailment Behaviour of an individual.

## 3 Research methodology

Our study was performed in three stages: (1) Scale Development, (2) Pilot Study and Exploratory Factor Analysis, and (3) Final Study. In the following, each of these stages will be elaborated upon in greater detail.

### 3.1 Stage 1: scale development

Taking a cue from the extant literature, factors like *financial concern, government policy, eco-concern, religiosity, and social norms* have been considered to play a significant role in determining curtailment behaviour. The scale items related to financial concerns have been adapted from the study by Karlin et al. (2012). The scale to measure environmental concern has been adapted from the work of Khan and Kirmani (2018). The items to measure religiosity have been adapted from the study of Koku and Jusoh (2014), whereas the items on the factor of social norms have been adapted from the study of Hwang (2009). Moreover, the scale items of the curtailment behaviour have been adapted from the work of Testa et al. (2016). As we failed to find a standard scale to measure the factor of government policy, we had to develop the factor items (Table 1). The scale items were developed taking cues from different policies the government has formulated to promote safeguarding the environment. The questionnaire items measuring all the constructs were based on a *5-point* Likert scale (1 = *strongly disagree, 5 = strongly agree*).

**Table 1:** Items and EFA result.

Item code	Item	Loading	Factor
**Total variance = 68.44 percent; KMO = 0.897; BTS (sig = 0.000)**			
SOCIAL1	My family thinks that I should not waste energy	0.727	**Social norms** Cronbach’s Alpha = 0.888
SOCIAL2	People around me encourage me to think about the environment	0.826	
SOCIAL3	My friends motivate me to change my consumption pattern	0.755	
SOCIAL4	The society we live in encourages curtailment behaviour	0.786	
ECOCON1	The population must act in an environmentally conscious way	0.692	**Eco-concern** Cronbach’s Alpha = 0.872
ECOCON2	We should be concerned about the environmental conditions under which we live	0.644	
ECOCON3	We must take measures to protect the environment as environmental catastrophe is a threat which needs attention	0.780	
ECOCON4	We must check our lifestyle for the benefit of the environment	0.641	
ECOCON5	To avoid any environmental disaster, I would restrain my consumption pattern	0.686	
CURT1	Keeping in mind the environment, I prefer public transport instead of personal	0.662	**Curtailment behaviour** Cronbach’s Alpha = 0.826
CURT2	I feel better in switching off appliances when not in use	0.723	
CURT3	I make sure not to waste any resources (water, electricity, energy etc.)	0.700	
CURT4	I avoid using single-use plastic	0.762	
REL1	I believe in the presence of the God	0.567	**Religiosity** Cronbach’s Alpha = 0.760
REL2	I live my life according to my religion	0.764	
REL3	My religious beliefs motivate me not to waste anything (electricity, water, food etc.)	0.607	
REL4	My religious teachings play a significant role in my decisions		
FINCON1	I pay close attention to my consumption pattern and monthly expenditures	0.752	**Financial concern** Cronbach’s Alpha = 0.768
FINCON2	I would love to save money on my bills	0.754	
FINCON4	When purchasing household appliances, I pay attention to their running costs	0.654	
GOVPOL1	Government policy is important in tackling environmental-related issues	0.692	**Government policy** Cronbach’s Alpha = 0.695
GOVPOL2	Government should invest more to prevent environmental catastrophe	0.713	
GOVPOL3	I keep track of and follow environmentally friendly policies initiated by the government	0.645	
GOVPOL4	Government policies impacts the consumption pattern of the consumers	0.577	
**Items deleted due to low loadings**			
FINCON3	I prefer public transport because of financial benefit	<0.40	**Deleted**
SOCIAL5	The media frequently suggest to us to curtail usage	<0.40	**Deleted**

KMO, Kaiser-Meyer-Olkin; BTS, Bartlet’s test of sphericity.

### 3.2 Stage 2: pilot study and exploratory factor analysis

To check the unidimensionality of the study constructs, EFA was employed on a small sample of 100 respondents (Hair et al., 2010). EFA is primarily employed to extract the maximum common variance and to arrange observed variables (the questionnaire items) in a common factor (also known as the latent variable) (Gorsuch, 2014). The correlation between the observed variable and the latent variable is expressed by factor loadings that range from 0 to 1. An observed variable can be attributed to a latent variable if the factor loading is greater than 0.4 (Cutillo, 2019).

There are various factor extraction techniques in factor analysis. Among them, Principal component analysis (PCA) was employed in our study. PCA forms uncorrelated linear combinations of the observed variables in such a way that the first component has maximum variance, and the successive components explain small portions of the variance. PCA gives only the initial factor solution and can be for a singular correlation matrix. For a more defined factor solution, Varimax rotation and Kaiser Normalisation were employed and used for factor extraction. Varimax is an orthogonal method of rotation that ensures that each factor has large loadings (greater than 0.4) for some observed variables and smaller loadings (less than 0.4) for other observed variables (Abdi et al., 2003). Thus, after the varimax rotation, a well-defined factor solution is observed wherein each latent variable is represented by a set of observed variables.

In this study, 6 items were extracted and identified as social norms, eco-concern, curtailment behaviour, religiosity, financial concern and government policy. The total variance explained was 68.44 percent. The Kaiser–Meyer–Olkin (KMO) value was 0.897 and Bartlett’s test of Sphericity (BTS) was significant. The Cronbach’s alpha values for the six constructs were 0.888 (social norms), 0.872 (eco-concern), 0.826 (curtailment behaviour), 0.760 (religiosity), 0.768 (financial concern), and 0.695 (government policy). All six factors were retained, but one item each of financial concern and social norms had to be removed because of low factor loadings (Table 1).

### 3.3 Stage 3: final study

#### 3.3.1 Sample and data collection

The Youth Policy in India delineates the youth demographic as individuals aged between 15 and 29 years. Consequently, data collection efforts were focused on individuals within this specified age group. Given the widespread internet usage among young consumers globally (Ahmad, 2020), data was primarily collected through online platforms. In India, approximately 65 per cent of internet users are within the youth demographic (Distribution of Internet Users in India 2013–2025, by Age Group, 2022), which underscores the appropriateness of online data collection for this study. This approach also ensured the inclusion of participants from diverse regions across the country. Drawing on the methodology adopted by Bashir and Madhavaiah (2015), the research instrument used in this study (a closed-ended structured questionnaire) was prepared in Google Docs. The online version of the questionnaire was uploaded on various online platforms like LinkedIn, Instagram, and Facebook. It was also sent to various groups and individuals through email, WhatsApp, Telegram, and Messenger to get more responses. The respondents participating in the survey were notified that their involvement was both voluntary and confidential. The survey remained active for data collection for 30 days. The survey resulted in the generation of data from 513 respondents. Of these, 35 questionnaires were unfit for further analysis and had to be eliminated. Thus, we ended up with 478 responses fit for further analysis.

## 4 Results

### 4.1 Confirmatory factor analysis

CFA was employed with the twin objectives of checking the goodness of fit of the measurement model and assessing the construct validity of the research instrument (Hair et al., 2010). CFA was performed on the six constructs. The item loadings for some items were low, and hence these items were removed (REL4 and GOVPOL4). The model fit indices were acceptable (Minimum Discrepancy Function by Degrees of Freedom divided (CMIN/df) = 2; Tucker-Lewis Index (TLI) = 0.884; Comparative Fit Index (CFI = 0.901); Standardized Root Mean Squared Residual (SRMR) = 0.0714; Root Mean Square Error of Approximation (RMSEA) = 0.0.081) (Hair et al., 2010).

The construct validity of the research instrument was evaluated based on three parameters. These are Composite Reliability (CR), Convergent Validity (CV), and Discriminant Validity (DV). CR refers to the extent to which a scale is accurately measuring the underlying constructs. CR values of 0.7 or more denote reliability (Fornell and Larcker, 1981; Hair et al., 2010; Malhotra and Dash, 2011). The CR values were greater than 0.7 for all the constructs.

The CV refers to the degree of convergence of different observed variables that are supposed to measure the same construct. The CV is assessed using the value of average variance extracted (AVE). AVE greater than the value of 0.5 indicates that an observed variable represents well a latent construct (Hair et al., 2010). The values for *AVE* were acceptable for all constructs (see Table 2), confirming convergent validity.

**Table 2:** Validity and reliability.

	CR	AVE	MSV	Social norms	Eco-concern	Curtailment behaviour	Religiosity	Financial concern	Government policies
Social norms	0.890	0.669	0.512	**0.818**	–	–	–	–	–
Eco-concern	0.878	0.592	0.538	0.715	**0.769**	–	–	–	–
Curtailment behaviour	0.856	0.603	0.538	0.660	0.734	**0.776**	–	–	–
Religiosity	0.894	0.739	0.333	0.427	0.577	0.536	**0.860**	–	–
Financial concern	0.788	0.558	0.278	0.527	0.474	0.434	0.205	**0.747**	–
Government policies	0.768	0.530	0.210	0.235	0.458	0.386	0.295	0.289	**0.728**

CR, Composite Reliability; AVE, Average Variance Extracted; MSV, Maximum Shared squared variance. Bold values means discriminant validity (diagonal values show the square root of average variance extracted).

The DV, on the other hand, represents the degree to which the different constructs are truly different from each other. DV was evaluated by comparing the values of AVE and Mean Shared Variance (MSV) (Fornell and Larcker, 1981; Hair et al., 2010). For all the constructs, the values of AVE were observed to be greater than the MSV values. The square root of AVE values on the diagonal (highlighted in bold) is greater than the inter-construct correlations in their respective columns (see Table 1). The Henseler et al. (2015) method of Heterotrait-Monotrait ratio (*HTMT*) was also used to assess discriminant validity. *HTMT* scores for all constructs were calculated using the plugin provided by Gaskin (2019) for AMOS 24.0. The *HTMT* scores for all the constructs were observed to be less than 0.85 (Table 3), thus confirming discriminant validity.

**Table 3:** HTMT scores.

	Social norms	Eco-concern	Curtailment behaviour	Religiosity	Financial concern	Government policies
Social norms	**–**	–	–	–	–	–
Eco-concern	0.732	**–**	**–**	–	–	–
Curtailment Behaviour	0.615	0.708	**–**	–	–	–
Religiosity	0.519	0.662	0.605	**–**	-	–
Financial concern	0.565	0.508	0.410	0.257	**–**	–
Government policies	0.213	0.409	0.383	0.287	0.271	**–**

### 4.2 Common method bias

The common method bias was assessed using the method of the common latent factor (CLF). The difference between standardised weights with and without CLF was less than 0.200 for all the constructs, ensuring the non-existence of a common method variance issue with the responses (Archimi et al., 2018).

### 4.3 Structural model

The values for the results of the hypothesis testing are presented in Table 4. The structural model findings showed that the construct of social norms significantly and positively affects curtailment behaviour (ß = 0.268; sig = 0.05). Hence, the hypothesis (H2) was supported. Similarly, eco-concern was observed to positively influence curtailment behaviour (ß = 0.383; sig = 0.05). Hence, the hypothesis was supported (H4). Religiosity was also observed to positively influence curtailment behaviour (ß = 0.165; sig = 0.05). Therefore, the hypothesis (H3) was also supported.

**Table 4:** Results of hypotheses testing.

Hypothesis code	Path	Estimates	C.R	Result
H1	Financial concerns → Curtailment behaviour	0.054^#^	0.836	Not supported
H2	Social norms → Curtailment behaviour	0.268^*^	**3.152**	**Supported**
H3	Religiosity → Curtailment behaviour	0.165^*^	**2.666**	**Supported**
H4	Eco-concerns → Curtailment behaviour	0.383^*^	**3.848**	**Supported**
H5	Government policy → Curtailment behaviour	0.083^#^	1.332	Not supported

* Significant <0.05. #Relationship not significant. C.R, Critical Ratio.

However, significant evidence was not observed for the influence of financial concern (ß = 0.054; sig > 0.05) and government policy (ß = 0.083; sig > 0.05) on the curtailment behaviour of the consumers. Hence, the hypotheses (H1 and H5) were not supported. Model fit indices for the final structural model were within the acceptable range (CMIN/df = 2.798; CFI = 0.903; TLI = 0.884; SRMR = 0.056; RMSEA = 0.081).

### 4.4 Moderating role of government policy

**Figure 2:**
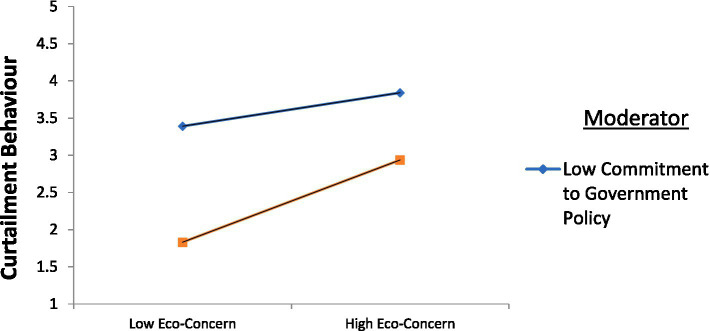
Moderating effect of government policy.

Next, the factor scores were calculated using the imputed technique in the CFA model. The aim of calculating factor scores was to examine the moderating effect of government policy on the relationship between economic concern and curtailment behaviour. The model was run with the interaction terms. The interaction terms represent the combined effect of two different variables, in addition to their individual effects on the dependent variable. In the current study, the interaction term was obtained by multiplying the study moderator ‘government policy’ with an independent variable ‘economic concern’. The findings suggest that the influence of the interaction term (Government policy **X** economic concern) on the curtailment behaviour is significant and positive (β = 0.117, *p* < 0.05). The model fit indices were within the acceptable range (CMIN/DF = 4.838, CFI = 0.989, TLI = 0.916, SRMR = 0.076, RMSEA =0.119). Hence, the hypothesis (H6) is supported. The impact of the interaction term is more clearly visible in Figure 2. It is clear from Figure 2 that the relationship between eco-concern and curtailment behaviour would be stronger for consumers exhibiting higher commitment to government policy than for consumers exhibiting low commitment to government policy.

## 5 Discussion

The escalation of environmental degradation has heightened consumer awareness about the issue. In a sense, they have joined hands to mitigate environmental issues. Consumers have turned towards ethical consumption, and thus their buying behaviour has changed. Researchers have suggested that the reduction of everyday carbon footprints by consumers can have a positive impact on the environment, but few studies have focused on the curtailment behaviour of consumers. Previous studies have mostly focused on green consumption patterns and have suggested several factors determining green consumption behaviour. Considering the importance of curtailment behaviour and aiming to identify the factors that influence consumers’ curtailment practices, we have tried to understand the underlying relationships between factors like financial concern, government policy, eco-concern, religiosity, and social norms on consumers’ curtailment behaviour.

Drawing on existing literature, we have endeavoured to evaluate the influence of various factors like financial concern, government policy, eco-concern, religiosity, and social norms play in determining the curtailment behaviour of consumers. We could hardly find studies considering government policy as a factor determining sustainable behaviour. Thus, government policies have been included as a factor determining curtailment behaviour in this study. Our study is novel in the context of focusing on curtailment behaviour. For this purpose, we have adapted a 26-item, six-factor scale.

The structural equation modelling revealed that only three factors, i.e., social norms, religiosity, and eco-concerns, have a significant impact on curtailment behaviour. The other two factors, financial concern and government policy, have no significant impact on curtailment behaviour.

Despite the assertion by numerous researchers (Testa et al., 2016; Huang et al., 2018) that financial concerns play a crucial role in green consumption and the willingness to pay for eco-friendly products, our results diverge from these earlier findings. The government encourages sustainable behaviour through the usage of energy-efficient products and offers huge subsidies for environmentally friendly products. It has also been observed that the tariffs imposed by the government have a considerable impact on domestic energy conservation. However, this study’s findings suggest that there is no impact of government policies on curtailment behaviour (H5). Several researchers (Kim et al., 2012) have indicated that social norms are a significant determinant of pro-environmental behaviour. Consistent with these earlier works, the results of our study also demonstrate a positive and significant influence of social norms on sustainable behaviour. Several researchers have also highlighted the substantial influence of religion on consumer behaviour. The findings of this study indicate that religiosity significantly impacts curtailment behaviour, aligning with the conclusions of previous research (Jurdi-Hage et al., 2019; Abutaleb et al., 2020).

Young consumers are more eco-friendly than any other generation (Kingston and Paulraj, 2024), which is the population of the present study. Environmental concern has been suggested to be a significant variable in the pro-environmental literature. Several researchers have suggested that environmental concern significantly and positively influences the sustainable behaviour of consumers. The results of the current study align with those of prior research (Ahmed et al., 2021). Although there was no direct impact of government policies on curtailment behaviour (H5), the study findings suggest that government policies strongly moderate the relationship between eco-concern and curtailment behaviour (H6). The total energy consumption in India ranks 3rd (World Energy and climate Statistics—Yearbook 2022, n.d.). This indicates a critical situation for the country, underscoring an urgent need for government intervention. For example, implementing tax incentives, subsidies, and other measures could foster eco-friendly behaviours. The relationship between eco-concern and the attitude (here, curtailment behaviour) of a consumer is positively moderated by government policies. Therefore, it can be inferred that favourable government policies are likely to motivate the population towards sustainable behaviour, and the opposite is the case for policies that are not well-received.

## 6 Conclusion and policy implications

Curtailment behaviour warrants global attention and collective action to mitigate the effects of environmental degradation. It is imperative that we unite in this effort. Academicians, marketers, policymakers, and consumers need to work together to imbibe curtailment habits among people. Academics can play a pivotal role in raising awareness about the necessity and adoption of curtailment behaviour through their research. This process can begin by detailing the global challenges arising from the excessive consumption of resources. Secondly, state the impact of the same on their lives. Finally, provide them with a solution.

The findings indicate that environmental concerns influence curtailment behaviour among individuals, highlighting the importance of disseminating information from an environmental perspective. It is suggested that consumers who begin to adopt eco-friendly behaviours do so gradually, as this approach can facilitate ongoing improvement both for themselves and for society at large.

Our study also contributes to broadening the theory-based insights available in this area. This study builds upon existing knowledge by uncovering the significance of financial concern, government policy, eco-concern, religiosity, and social norms in the context of curtailment behaviour. Notably, government policy and financial concern are two variables that have rarely been used in studies related to sustainable consumption behaviour or curtailment behaviour. The current study has also offered a new outlook by revealing that government policy through eco-concern indirectly impacts the curtailment behaviour of consumers. It can be surmised that mere concern about the environment by itself does not drive consumers to curtail; at times, good government policies make the consumers more eco-conscious and behave sustainably.

It is high time that policymakers think critically and find ways to motivate consumers to behave more sustainably. Given the current and anticipated stresses on our planet, everyone must come together for a singular cause: to make our planet sustainable for future generations. Policymakers need to comprehend both curtailment behaviour and the factors influencing it. Therefore, the insights from this study can be invaluable for policymakers, potentially guiding them to foster a culture of curtailment within their constituencies. Drawing on the study’s findings, it is proposed that curtailment behaviour—a key aspect of pro-environmental actions—can be encouraged among stakeholders by emphasizing social norms, environmental concerns, and religiosity. Given that consumer curtailment behaviour is poised to benefit society, policymakers should concentrate on these three determinants.

The study’s results reveal that government policies play a moderating role between environmental concerns and curtailment behaviour. Thus, it’s recommended that specific actions be taken, like facilitating the switch from incandescent bulbs to LEDs through the UJALA (Unnat Jyoti by Affordable LEDs for All) scheme. Additionally, efforts should be made to consistently engage with consumers through various technological platforms to reinforce these behaviours. According to research undertaken by the Solar Energy Corporation of India (SECI), assuming the country’s demand profile continues unaltered, the installations might result in the curtailment of 50% of solar power by FY 2030 (Jain and Srivastava, 2020). This will educate the consumers about the current issues as well as the policies (e.g., subsidies, fines), which will motivate the consumers to adopt curtailment behaviour.

## 7 Limitations and future scope

While the study has provided valuable insights into the factors influencing consumer curtailment behaviour, there are limitations that could have impacted the findings: Notably, the research focused on young consumers in India, a developing country. To test the robustness of the study findings, future researchers may extend this study to a different geographical setting. Secondly, the data was gathered through a convenience sampling method, which may limit the ability to generalise the study’s findings. In future work, researchers could collect data from a more representative and large population. Further studies can also extend and improve the proposed model by incorporating other relevant factors derived from the literature, which may provide further insights into the curtailment behaviour of individuals.

## Data Availability

The raw data supporting the conclusions of this article will be made available by the authors, without undue reservation.
